# Halogenated Anesthetics Determination in Urine by SPME/GC/MS and Urine Levels Relationship Evaluation with Surgical Theatres Contamination

**DOI:** 10.1155/2014/753237

**Published:** 2014-02-27

**Authors:** Serena Indelicato, David Bongiorno, Sergio Indelicato, Leopoldo Ceraulo, Ernesto Tranchina, Giuseppe Avellone, Concetta Arcadipane, Filippo Giambartino

**Affiliations:** ^1^Dipartimento di Scienze e Tecnologie Biologiche Chimiche e Farmaceutiche (STEBICEF), Università Degli Studi di Palermo, Via Archirafi 32, 90123 Palermo, Italy; ^2^Centro Grandi Apparecchiature-UniNetLab, Università Degli Studi di Palermo, Via F. Marini 14, 90128 Palermo, Italy; ^3^CQRC Laboratory, A.O.U.P. “P. Giaccone”, Via del Vespro 129, 90127 Palermo, Italy; ^4^Dipartimento di Scienze per la Promozione della Salute e Materno Infantile “G.D'Alessandro”, Sezione di Medicina del Lavoro, Via del Vespro, 90127 Palermo, Italy; ^5^Dipartimento di Biopatologia e Biotecnologie Mediche e Forensi, Via del Vespro, 90127 Palermo, Italy

## Abstract

In this work, a new sensitive analytical method has been developed and evaluated for the determination of the most commonly used gaseous anesthetics, desflurane, sevoflurane, and this latter's hepatic metabolite hexafluoroisopropanol (HFIP) in the urine. In addition, an evaluation of anesthetics exposition on the urine levels of a small population of surgical operators has been performed and results are briefly discussed.

## 1. Introduction

Halogenated anesthetics are important risk factors in occupational medicine field. Indeed, while their short-term effects for acute exposure are well known [1, 2] and some fatal cases [2–5] have been reported, chronicle exposures to these xenobiotics are not studied yet, even if toxic effects (abortion and liver damage) are suspected in people involved in these jobs. In addition, scientific research is leading to the development of protocols in which the use of anesthetic mixtures reduces their concentration; on the other hand, these require more sensitivity and selective analytical methods for their determination.

Inhalation anesthesia is indeed a widely used method for inducing complete anesthesia in surgeries. Several collateral or secondary effects due to acute exposure to inhalatory anesthetics are reported in the literature [1, 2, 6], while little information has been collected on chronicle exposure to small amounts of these drugs. Nowadays, the most representative class of anesthetics used is perfluorinated molecules such as desflurane and sevoflurane. These drugs are very often employed in synergy with nitrogen dioxide and sometimes with other anesthetics in order to reduce doses and anesthetic induction times.

The surgical operators exposure to the drugs is today drastically reduced due to the use of efficient air recirculation and refreshing systems in the surgical theatres and the development of anesthesia protocols. Nevertheless, symptoms such as drowsiness, reduction of concentration, and lack of clarity are sometimes still reported from operators, requiring a more effective control on working areas in order to reduce risks directly or indirectly connected to the inhalatory anesthetics use.

In order to establish if an effective amount of anesthetic is inhaled from operators, a very sensitive technique is required to determine the concentration of anesthetic in surgical theatres since a relatively small xenobiotics concentration is required to improve the working conditions.

While first approaches to determine anesthetics were based on hexane extraction [7, 8], more recently, gas chromatography (GC) equipped with different kinds of detectors has been used to identify these drugs [9–14]. The most recent method relies on rapid determination of sevoflurane and hexafluoroisopropanol (free, unconjugated form) based on direct injection of human plasma into GC/MS apparatus without any sample clean-up procedures [15]. Indeed, GC/MS technique is nowadays a very common analysis tool and its field of application can be extended to moderately polar to very polar molecules by appropriate derivatization/modification procedures [16, 17].

In our work, a very sensitive method based on solid phase microextraction gas chromatography mass spectrometry for determination of anesthetics in urine has been developed.

The analysis results were also evaluated in the light of independent environmental anesthetics determinations obtained through a complementary technique based on the photo-acoustic detection principle [18].

**Figure 1:**
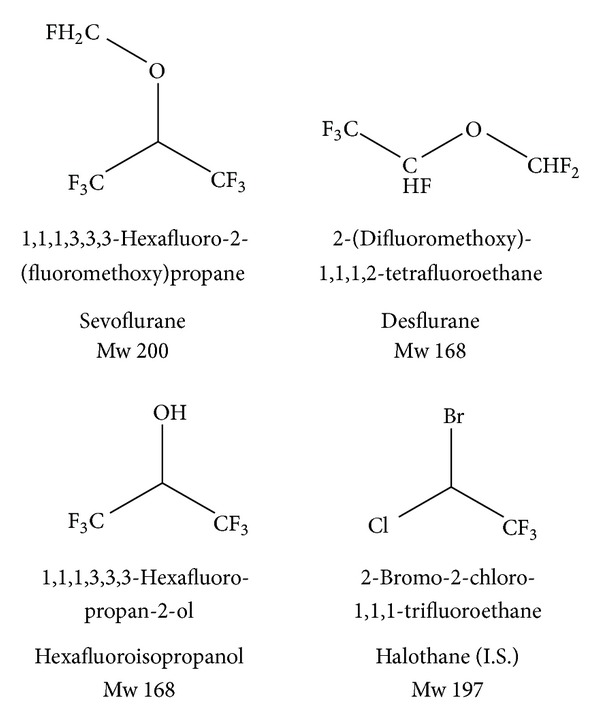
Structures of halogenated anesthetics determined sevoflurane and desflurane, of sevoflurane's metabolite hexafluoroisopropyl alcohol, and of halothane (internal standard).

In particular, sevoflurane, its metabolite hexafluoroisopropyl alcohol, and desflurane were taken into consideration (Figure 1), using halothane as internal standard and matrix matched calibration curves.

This approach took into consideration the matrix effect, which also, in our case, proved to be relevant especially when SPME was used. Due to its high sensitivity, rapidity, and the simultaneous determination of different anesthetics in urine samples, the present method represents a further development in comparison with the literature [19–21].

## 2. Experimental

### 2.1. Reagents and Materials

Water (HPLC grade), methanol (HPLC grade), *β*-glucuronidase from bovine liver (≥1,000,000 units/g solid), and sodium chloride were purchased from Sigma-Aldrich (Supelco, Bellafonte).

SPME experiments were carried out using three kinds of coated fibers: 100 *μ*m PDMS, 65 *μ*m PDMS/CAR, and 50/30 *μ*m DVB/CAR/PDMS. These fibers were purchased from Sigma-Aldrich (Supelco, Bellafonte).

### 2.2. Standards, Internal Standards, and Stock Solutions

Sevoflurane (99,9%) and desflurane (99,8%) were purchased from Baxter (Deerfield, IL); halothane (99,5%) and hexafluoroisopropyl alcohol (99%) were purchased from Sigma-Aldrich (Supelco, Bellafonte). Standard solutions preparation was made according to this procedure: 20 *μ*L aliquots of sevoflurane, desflurane, and hexafluoroisopropanol (HFIP) were weighed and dissolved in methanol in 100 mL flasks. Due to their high volatility, for all analytes (sevoflurane, boiling point: 331.75 K at 101.3 kPa and density 1.5 × 10^3^ kg/m^3^ at 293 K; desflurane, boiling point: 296.65 K at 101.3 kPa and density 1.5 × 10^3^ kg/m^3^ at 293 K; HFIP, boiling point: 332 K at 101.3 kPa and density 1.6 × 10^3^ kg/m^3^ at 293 K), pure standards volumes were picked through cold (255 K) tips and were rapidly weighed into volumetric flasks containing 10 mL of cold methanol.

These solutions were then diluted with a further amount of cold methanol (255 K) up to the mark (100 mL) at ambient temperature (298 K).

The solutions obtained had a final concentration of 302 *μ*g mL^−1^ for desflurane and sevoflurane and 320 *μ*g mL^−1^ for HFIP. These stock solutions, stored in refrigerator at 277 K ± 2 K, were checked weekly and were found to be stable in these conditions up to three months.

An aqueous mother solution containing all the analytes at a concentration of 250 *μ*g L^−1^ has been prepared using these standard solutions and it has been found stable for no more than a month. Further diluted solutions, used to make the matrix matched calibration curves, were prepared weekly by simple dilution of the mother solution in HPLC grade water.

Internal standard solution was prepared picking 20 *μ*L of halothane standard (density 1,86 × 10^3^ kg/m^3^ at 293 K) through cold (255 K) tips and rapidly weighing into volumetric flasks containing a small amount of cold methanol (about 10 mL at 293 K).

The obtained solution was then further diluted up to the mark (100 mL) with methanol at ambient temperature (298 K). The solution obtained has a final concentration of 380 *μ*g mL^−1^ and was stored in refrigerator at 277 K ± 2 K. This solution was checked weekly and it was found to be stable in these conditions up to three months.

### 2.3. Apparatus

Analyses were carried out using Focus GC Thermo equipped with a capillary column Supel Q-Plot (30 m × 0.32 mm I.D. × 15 *μ*m average thickness), coupled with a DSQ II Mass Spectrometer, working in selected ions monitoring (SIM) mode. The analysis method was optimized in order to reach the maximum efficacy in terms of rapidity, selectivity, and sensitivity.

**Figure 2:**
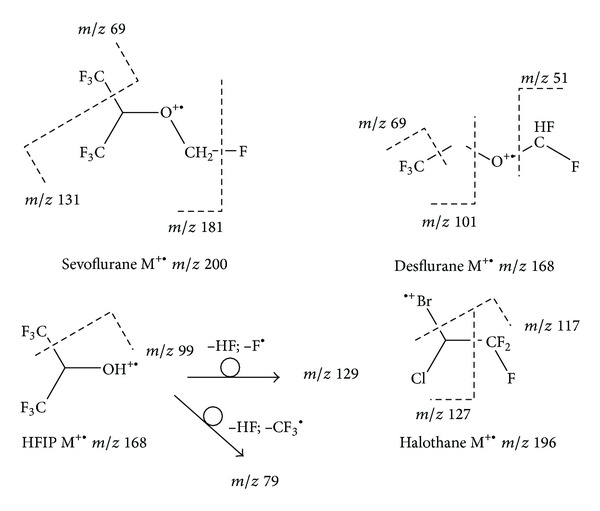
Rationale of the fragmentation processes explaining the selected peaks for the SIM analysis.

The best trade-off obtained leads to a total analysis time of 13 minutes (see Figure 2). Operating conditions were the following: injector temperature, 473 K; splitless (gas flow 1 mL min^−1^, splitless flow for 1 min with a gas flow 10 mL min^−1^); constant flow 1 mL min^−1^; and carrier gas, helium. The thermal program starts at 309 K and holds for 3 min and then the temperature increases at 40 K min^−1^ up to 403 K and holds for 7 minutes at this temperature.

According to the literature and the experimental data obtained from pure standards full scan MS spectra, the most representative mass peaks have been chosen (see Table 1).

These ions were used in SIM mode in order to increase sensitivity, with respect to full scan MS.

The rationale fragmentation processes that could explain these peaks are briefly reported in Figure 2.

### 2.4. Samples Preparation

The following procedure was applied for the real samples preparation: 18 mL sealed vials were prepared with 1 mL of enzyme solution (*β*-glucuronidase 999 Ui/mL), 10 *μ*L of acetic acid, 40 *μ*L of internal standard solution, and 2.0 × 10^−3 ^kg of NaCl. 3950 *μ*L of surgery's operator urine was added by means of gastight syringe into the previously prepared vials. In order to avoid loss in IS concentration, the urine volumes were placed into the vials, without unscrewing the cap and piercing the septum with the syringe's needle. After having placed the vials at 310 K for 16 h to obtain the enzymatic hydrolysis of the glucuronate-HFIP, the samples were analyzed.

Matrix matched calibration samples were prepared weekly according to the following procedure: 1 mL of enzyme solution (*β*-glucuronidase 999 Ui/mL), 10 *μ*L of acetic acid in order to generate the optimal conditions (pH 4-5) for the enzyme activation, 40 *μ*L of internal standard solution, 1 mL of standard solution containing the mix of anesthetics at increasing concentration, and an aliquot of urine (pooled 50% from female and 50% from male voluntary healthy subjects) up to final volume of 5 mL were added in a 18 mL vial, sealed with PTFE/silicon septum; finally, in order to get a salting-out effect, 2.0 × 10^−3 ^kg of NaCl was added. For the sake of strict repeatability, standards were subjected to 16 hours of incubation at 310 K (normally necessary for the enzymatic hydrolysis of the glucuronate-HFIP) before analysis.

### 2.5. Solid Phase Microextraction Procedures (SPME)

**Figure 3:**
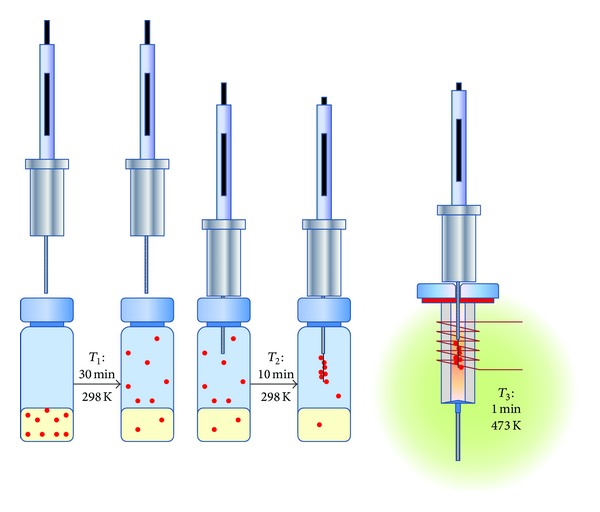
SPME sampling procedure scheme.

SPME technique required a preliminary study to determine the most straightforward extraction procedure. The final optimal conditions were the following: fiber DVB/CAR/PDMS; equilibration time (*T*_*1*_): 30 minutes (298 K ± 1 K); extraction time (*T*_*2*_): 10 minutes (298 K ± 1 K). The best fiber desorption conditions were the following: injector at 473 K, desorption time (*T*_*3*_) of 1 minute (splitless mode). See Figure 3.

### 2.6. Environmental Monitoring and Real Samples Collection

Anesthetic concentrations in different selected operating rooms were measured by Multi-Gas monitor Type 1302 (Brüel & Kjær, Denmark), whose design was based on the photo-acoustic detection principle. This instrument is capable of continuously recording the gaseous concentrations of fluorinated anesthetics within the surgery room.

More than 25 real samples were collected from operators working in three different surgical rooms. Analyses were carried out in duplicate on pre- and post shift urine samples of both the operators coming in contact with the anesthetics during their shift (i.e., surgeons or anesthetists) and the ones that are not usually in contact with xenobiotics during shift (i.e., medical attendants).

## 3. Results and Discussion

### 3.1. Method Optimization

Solid phase microextraction is a sample preparation method, invented by Pawliszyn in 1989, which does not require solvents. Since its first applications to environmental and food analysis, its use is becoming wider and frequent, due to its environmentally friendly features, effectiveness, and reduced costs. The principle of SPME is based on a fused-silica fiber that is coated with an appropriate stationary phase. All analytes in the sample are directly extracted to the fibre coating, in a single step that involve both extraction and concentration from the headspace of the vial.

**Figure 4:**
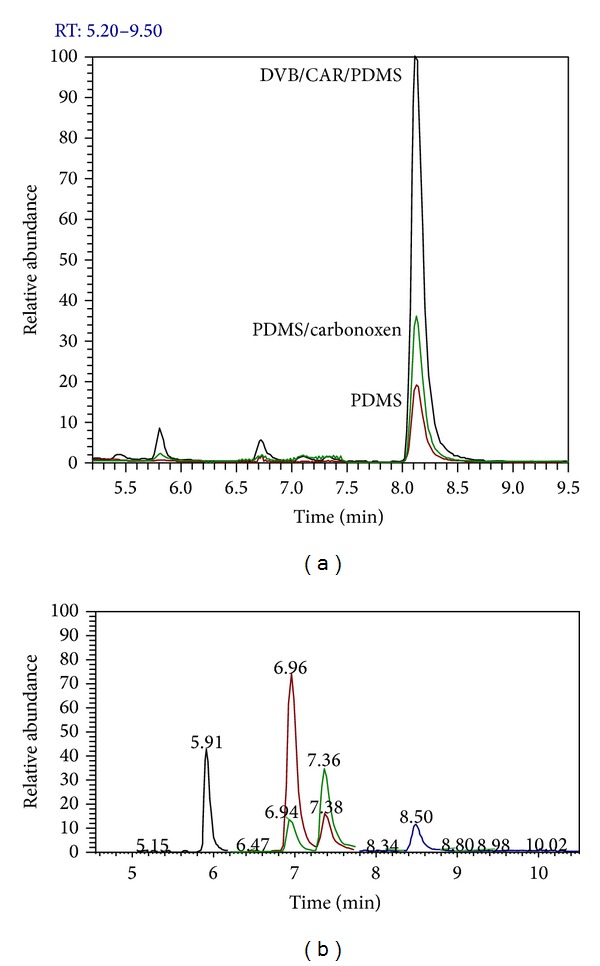
Internal standard (halothane) peaks obtained with three different types of fibers. The most efficient extraction has been obtained through DVB/CAR/PDMS fiber.

Because of the high volatility of the compounds, optimizing the efficiency and sensitivity of the analysis method is necessary to identify the most suitable SPME sampling conditions. The first step in the development of a SPME method was the fiber selection. Therefore, three different SPME fibers (PDMS; PDMS/CAR; DVB/CAR/PDMS) have been tested. The analyte peak areas, obtained from analyses of standard solutions at the same concentration of anesthetics, were compared and the higher efficiency of extraction of all the analytes was reached using the DVB/CAR/PDMS fiber (according also to Poli et al., 1999). See Figure 4.

The second step was the determination of the optimal saturation time, needed for the analytes to reach equilibrium between the matrix, the headspace, and the fiber. The headspace sampling was performed at the same temperature (298 K ± 1 K) for all the analytes and optimal saturation time was determined; the analyte peak areas after 15, 30, and 60 minutes of equilibration were compared, showing that 30 minutes were enough to reach equilibrium in headspace. Finally, in order to improve the extraction efficiency, various extraction times were evaluated. The comparison between the responses obtained from extraction times of 5, 10, 20, and 30 minutes, at the same temperature (298 K ± 1 K), indicates that after 10 minutes the extraction efficiency reached a plateau.

No further raise in analyte peak areas resulted from increase of the fiber exposition time in HS.

### 3.2. Calibration Curves and Method Performance Evaluation

**Figure 5:**
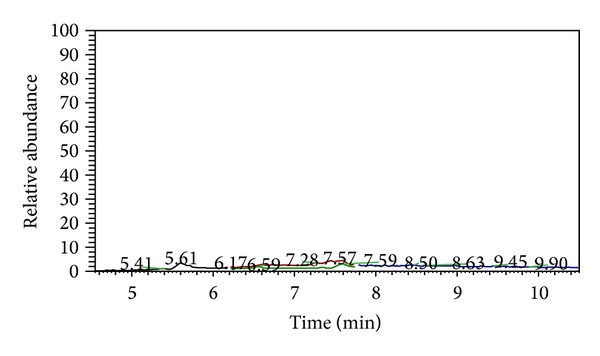
GC/MS chromatogram of fortified urine at 5 *μ*g L^−1^ (a) and blank (b) urine.

A preliminary calibration curve was made in water in order to achieve the order of magnitude of both limit of detection (LOD) and limit of quantification (LOQ) and to verify the linearity range of the whole method without any interference due to matrix. These provided a very strong linear correlation (*R* ^2^ > 0.99) in a range from 0.32 *μ*g L^−1^ to 40 *μ*g L^−1^. GC/MS chromatogram of 5 *μ*g L^−1^ fortified (a) and blank (b) urine are reported in Figure 5 to show analytes separation and method sensitivity in matrix.

The effect of the matrix has been evaluated in order to verify its eventual impact on linearity, sensitivity, and slope of the calibration curves using matrix matched calibration curves to determine effects of the matrix at different levels of concentration.

**Figure 6:**
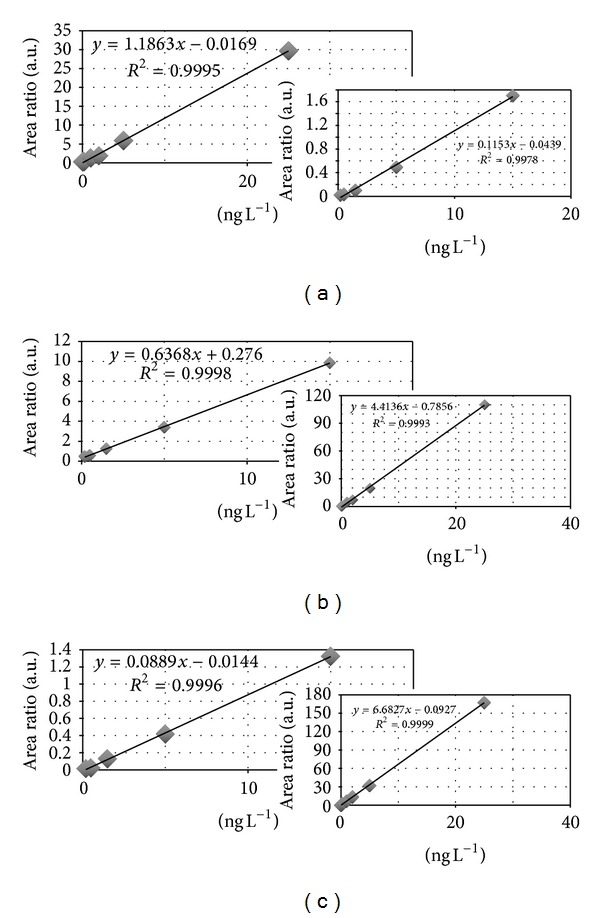
Matrix matched calibration curves for desflurane (a), sevoflurane (b), and HFIP (c) in the insert are reported as the corresponding curves in water.

Indeed SPME analysis is quite prone to matrix effect repercussions on calibration curves. In addition, in our experience, the intrinsic low reproducibility of precise experimental conditions (i.e., intrinsic sample differences, fiber wearing, etc.) often requires an internal standard to effectively reduce errors. When a matrix effect is observed, as in this case, both a matrix matched calibration curve and an internal standard should be used in order to reduce errors and improve the accuracy of the measures. As shown in Figure 6, a marked matrix effect is observed when determining the same anesthetic concentration in water and urine. Indeed, very different (sample/internal standard) area ratios are observed looking at “calibration curves” obtained in distilled water (inserts) and calibration curves made using pooled urine as matrix. This matrix effect markedly lowers the calibration curve slopes (and consequently the sensitivity) with respect to calibration curves obtained in pure water. This defeats any attempt to use an external calibration and requires a matrix effect correction. However, matrix matched calibration curves repeated using different urine pools at distance of one to several weeks demonstrate good reproducibility, with similar slopes and intercepts. This demonstrates that matrix effect is not peculiar for each sample and does not require more complicated and time consuming multiple standard addition procedures in order to get reliable measures.

The linearity of the method using matrix matched standards remains fair, even if it has been ascertained in a range of concentration that is smaller (from 0.30 *μ*g L^−1^ to 22.5 *μ*g L^−1^ for sevoflurane and desflurane and in the range 0.32 *μ*g L^−1^ to 24 *μ*g L^−1^ for HFIP) than the corresponding linearity obtained in water solution (from 0.06 *μ*g L^−1^ to 37.5 *μ*g L^−1^ for sevoflurane and desflurane and in the range 0.6 *μ*g L^−1^ to 40 *μ*g L^−1^ for HFIP; see Figure 6) standards.

The three curves obtained were weighed on 1/*x* in order to enhance the curve fitting at a lower concentration and show a very good coefficient of correlation *R* ^2^ (see Table 2).

Also LOD and LOQ have been calculated according to this procedure: average and standard deviation values of at least 20 blanks have been reckoned and LOD and LOQ values are defined as blanks signal average plus 3 and 10 times the standard deviation of the blanks, respectively.

The following performances studies were, moreover, carried out: within-laboratory repeatability (WLr) and within-laboratory reproducibility (WLR) expressed as % RSD on sample at 5.0 *μ*g L^−1^ (see Table 2).

### 3.3. Cases Study Results

The extended performances reached through this method were useful to evaluate the anesthetic exposure effects on a small number of operators working in surgical theaters. In the selected theaters, a parallel evaluation of the environmental instantaneous levels of anesthetic was also conducted in order to evaluate eventual relationships between the “environmental pollution” and the anesthetics biological levels. The observed correlation was indeed scarce and no correlation was found between the exposition time and the anesthetics biological levels. Actually, it emerged that, during the surgical operations,the staff exposition time to anesthetics is largely variable and not linked to the working hours. Another exposition factor such as the distance between the vaporizer and each individual is variable as well. Thus, a correlation between environmental pollution and urine levels should not be expected. However, a variation in urine levels of anesthetics has been definitely ascertained.

**Figure 7:**
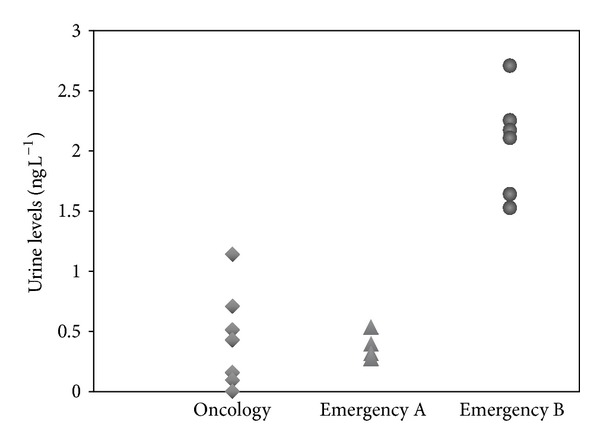
Spread of urinary level increments of desflurane for the different surgery rooms (□ squares: oncology; ∆ triangles: emergency A; ○ circles: emergency B).

The samples collected from operators working in three different surgical theaters were used to determine desflurane levels, while those collected from the other two surgical theaters were employed to determine the sevoflurane levels and its metabolite HFIP in urine. It is worth noting that, in all cases, the anesthetics levels determined after shift were higher than the corresponding levels in pre-shift samples. In particular, high levels of desflurane anesthetic were determined in people working in emergency B surgical theaters. From anamnesis, it was ascertained that several people mostly working in this theatre reported symptoms such as headache, drowsiness, asthenia, and concentration diseases, while the same diseases were rarely found in people working in the other theaters. Urine levels can be taken as a clue of the air condition system efficiency being mean levels of anesthetics well grouped for each surgical theatre; see Figure 7. It is also interesting to note that levels before and after shift were in the *μ*g L^−1^ order, mostly corresponding to the LODs of the previously reported methods [19, 20]. This implies that, in most cases, up to now, only few positive cases could be detected, of subject being exposed to anesthetics. From our determinations, instead, it seems that, even if in different extents, exposition to anesthetics involves all the personnel working in the theatres. Indeed, post-shift urine mean levels were always significantly higher than pre-shift ones.

## 4. Conclusions

The developed analytical method proved to be very quick, sensitive, and robust (precision, calculated as RSD, was always below 13% for all intra- and interday determinations). Sample preparation is simple and can be performed even by not highly specialized personnel.

It represents a further development in comparison with the literature being the first method for the quantification of desflurane in biological fluids. Indeed, this method exhibits LOD and LOQ values at least 2 magnitude orders lower than those previously reported [19–21] for similar volatile anaesthetics. This is probably due to the synergism of a well-optimized SPME procedure and GC/SIM-MS analysis. The preliminary data obtained by this pilot study on surgical theatres stressed the effectiveness of this method as a useful tool to monitor the exposition to anaesthetics in occupationally exposed people.

Indeed, with this method, it has been possible to ascertain significant difference between the anesthetics levels in urine before and after the work shifts in 25 individuals. Further, significant differences in post-shift urine levels have been found in personnel working in different surgical theaters. This means that different working conditions and probably differences in efficiency of the environmental treatment effectively reflect on personnel exposition to these xenobiotics.

## Figures and Tables

**Table 1:** Selected ions for SIM analysis.

Anesthetic	Selected ions (*m/z*)
Desflurane	51; 69; 101
Sevoflurane	69; 131; 181
HFIP	79; 99; 129
Halothane	117; 127; 196

**Table 2:** Method performances.

Analyte	LOD (*µ*g L^−1^)	LOQ (*µ*g L^−1^)	*R* ^2^ (weighed 1/*x*)	WLr	WLR
Desflurane	0.015	0.022	0.996	9.90%	11.50%
Sevoflurane	0.022	0.039	0.994	11.60%	12.47%
HFIP	0.024	0.042	0.996	2.71%	14.22%
